# Classification and Analysis of Human Body Movement Characteristics Associated with Acrophobia Induced by Virtual Reality Scenes of Heights

**DOI:** 10.3390/s23125482

**Published:** 2023-06-10

**Authors:** Xiankai Cheng, Benkun Bao, Weidong Cui, Shuai Liu, Jun Zhong, Liming Cai, Hongbo Yang

**Affiliations:** 1 School of Biomedical Engineering (Suzhou), Division of Life Sciences and Medicine, University of Science and Technology of China, Hefei 230026, China; chengxk@sibet.ac.cn (X.C.); bbk21468@mail.ustc.edu.cn (B.B.); shliu2000@mail.ustc.edu.cn (S.L.); zhongj@sibet.ac.cn (J.Z.); 2 Suzhou Institute of Biomedical Engineering and Technology, Chinese Academy of Sciences, Suzhou 215163, China; cuiwd@sibet.ac.cn (W.C.); cailm@sibet.ac.cn (L.C.)

**Keywords:** acrophobia, virtual reality, body movement, machine learning, sensor network

## Abstract

Acrophobia (fear of heights), a prevalent psychological disorder, elicits profound fear and evokes a range of adverse physiological responses in individuals when exposed to heights, which will lead to a very dangerous state for people in actual heights. In this paper, we explore the behavioral influences in terms of movements in people confronted with virtual reality scenes of extreme heights and develop an acrophobia classification model based on human movement characteristics. To this end, we used wireless miniaturized inertial navigation sensors (WMINS) network to obtain the information of limb movements in the virtual environment. Based on these data, we constructed a series of data feature processing processes, proposed a system model for the classification of acrophobia and non-acrophobia based on human motion feature analysis, and realized the classification recognition of acrophobia and non-acrophobia through the designed integrated learning model. The final accuracy of acrophobia dichotomous classification based on limb motion information reached 94.64%, which has higher accuracy and efficiency compared with other existing research models. Overall, our study demonstrates a strong correlation between people’s mental state during fear of heights and their limb movements at that time.

## 1. Introduction

Human movement data are a valuable information resource. A number of studies have now revealed that human movement information can be used to assess the quality of human health and to classify and identify people’s daily activities. For example, inertial sensors were attached to the legs of infants to obtain motion acceleration data and angular velocity data to explore the relationship between motion complexity and developmental outcomes in infants at high familial risk for autism spectrum disorder (ASD) (HR infants) [1], and to develop a classification model between HR infants and normal infants. Statistical analysis of motor acceleration signals at the wrist and ankle in children with normal development and children with attention deficit hyperactivity disorder (ADHD) [2] suggests that the two have different statistical properties of behavior. With the help of motion data from inertial guidance sensors at the human wrist and ankle, or from the accelerometer data that comes with the mobile phone, direct analysis is performed and a classification model [3] is built to enable activity recognition [4,5] for everyday activities such as walking, running, and walking up and down stairs [6], as well as more in-depth gait analysis [7], fall detection [8], and biomedical information detection [9,10]. Further, six-axis inertial sensors [11] are attached to the head, left forearm and right forearm, as well as the left lower tibiae and right lower tibiae, to obtain motion information data and reproduce the human posture through a data network.

At the same time, human movement data in a particular environment can also be used to stimulate and reflect certain current psychological states, such as fear of heights. Acrophobia (fear of heights) is a psychological disorder that refers to an extreme fear and discomfort of heights, even to the point of pathology. People with acrophobia usually feel the fear of losing their balance or falling from high places—even when they are in the safety of a fence or protective barrier—and experience a strong sense of unease and fear. Research indicates that individuals with acrophobia exhibit significant differences in their motor patterns compared to those without acrophobia. Specifically, acrophobia patients tend to exhibit more cautious, defensive postures such as hunching and neck retraction in high places [12,13], while individuals without acrophobia stand or walk more relaxed, confident, and naturally. With the development of society, more and more skyscrapers have emerged [14], creating more opportunities for people to experience high altitude. However, for individuals suffering from acrophobia, this can be dangerous.

Therefore, it is important to assess and analyze individuals with acrophobia to help them understand the extent of their fear and receive personalized treatment when considered necessary. Currently, the mainstream assessment and treatment methods for acrophobia are cognitive-behavioral therapy and virtual reality exposure therapy. Cognitive-behavioral therapy involves face-to-face communication between a psychotherapist and the patient for assessment and treatment [15,16]. While this approach is effective, the one-to-one communication sessions will not only lead to overall inefficiency, but will mean that a large number of psychotherapists will be needed at a social level to focus on the problem; therefore, this approach will be too dependent on the professionalism of the therapist. The second method is virtual reality exposure therapy (VRET) [17], a behavioral therapy used for anxiety disorders, including phobias. Patients immerse themselves in a computer-generated virtual environment that can provide various scenarios that are difficult to create in the real world [18]. The environment allows for controlled safety measures that can be manipulated to meet the individual’s needs. As early as 1990, virtual reality technology was used in research related to phobias [19]. In recent years, more research on acrophobia has been based on virtual reality technology. Due to the expanded visual range, high-altitude environments tend to cause vertigo and affect people’s control of their posture [20,21,22]. It has been found that, from a power spectrum perspective, an increase in height in the virtual environment typically reduces low frequency (<0.5 Hz) body sway and increases oscillations in high frequency (>1 Hz) body sway in the frequency range 0–3 Hz, and this change is more pronounced in people who are truly afraid of heights [23]. In addition, the amplitude of body sway decreases with increasing virtual height [24]. This means that people who are afraid of heights and those who are not afraid of heights will have a different sense of motion pattern in an aerial environment.

In conclusion, we hope to investigate automated models for classifying and analyzing acrophobia by examining the movement patterns of acrophobic and non-acrophobic people at height, so that people can quickly and correctly recognize their acrophobic state. To achieve this, we designed common daily movement tasks (walking, ball retrieval, ball release, jumping) in a virtual reality altitude environment and used a set of easy-to-use, miniaturized wireless sparse sensor devices designed to acquire movement information at joints to characterize the movement of acrophobic and non-acrophobic humans under high-altitude conditions, and proposed a set of human movement-based acrophobic and non-acrophobic classification algorithm models, which can achieve efficient and fast classification.

## 2. Materials and Methods

This section centers on the wireless miniaturized inertial navigation sensor (WMINS) and the development of immersive virtual reality scenario, alongside the associated data collection and analysis classification process.

### 2.1. Wireless Miniaturized Inertial Navigation Sensor (WMINS)

**Figure 1:**
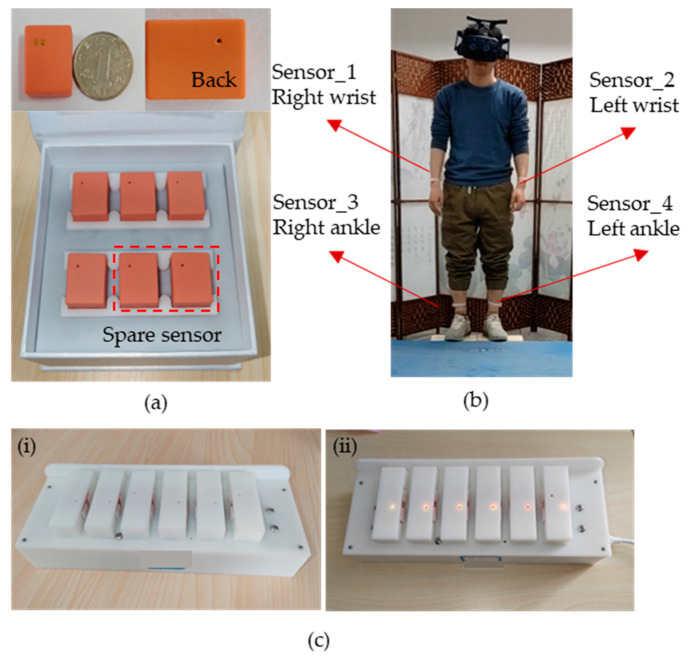
Introduction to wireless miniaturized inertial navigation sensor (WMINS). (**a**) Outline dimensions of the sensor and the introduction of the whole set of equipment. (**b**) Schematic diagram of the attachment position of the sensor to the human body. (**c**) External magnetic charging of the sensor. (**i**) No charging state, (**ii**), charging state.

As shown in Figure 1a, we have developed the WMINS sensor with a housing size of only 23.5 × 18.6 × 9.6 mm and a bare board size of 16.75 × 11.22 mm, the size of a common dollar coin in the market. The weight is amazingly light at 3.8 g, equivalent to a common dollar coin in the market. Due to its lightweight and small size, the WMINS sensor can be attached to any part of the human body to collect motion information without being restricted by joints. The sensor comprises two major functional modules—a motion collection module for collecting the motion information of the attached object and a data transmission module that uses low-power Bluetooth technology for data transmission and reception [25]. Furthermore, by designing a Bluetooth one-to-four wireless sensor network, four WMINS sensors can independently collect and transmit data simultaneously, thus constructing the motion collection system for key body parts. Figure 1b illustrates the specific usage mode of the WMINS sensor in human motion detection and collection. Four WMINS sensors are attached to the upper part of the left and right wrists and lower tibiae of the human body, which are key parts of the limbs, and data acquisition and transmission occur at 30 Hz. Therefore, motion information is obtained through these four joint parts. As the WMINS sensor is remarkably small, it does not require an additional touch switch, as the touch switch is already integrated on the back to save space. With just a light touch of two fingers, WMINS can be effortlessly turned on. Once the sensor is activated, the top left corner of the back will flash blue light at 1 Hz. When the sensor is connected to the receiving device, WMINS will display a steady blue light. Figure 1c showcases the charging mode of WMINS. We have designed a charging box that can accommodate multiple sensors for charging. To minimize the size of WMINS, we have adopted a spring probe charging mode, which incorporates a retractable spring into each charging port that matches the WMINS in Figure 1a. In order to reduce the weight of WMINS, a small circular magnet is placed externally on the portable charging box to fix the sensor in the charging state. The left image in Figure 1c shows the sensor in an uncharged state, and the right image shows the sensor in charging state, with WMINS displaying a yellow light.

**Figure 2:**
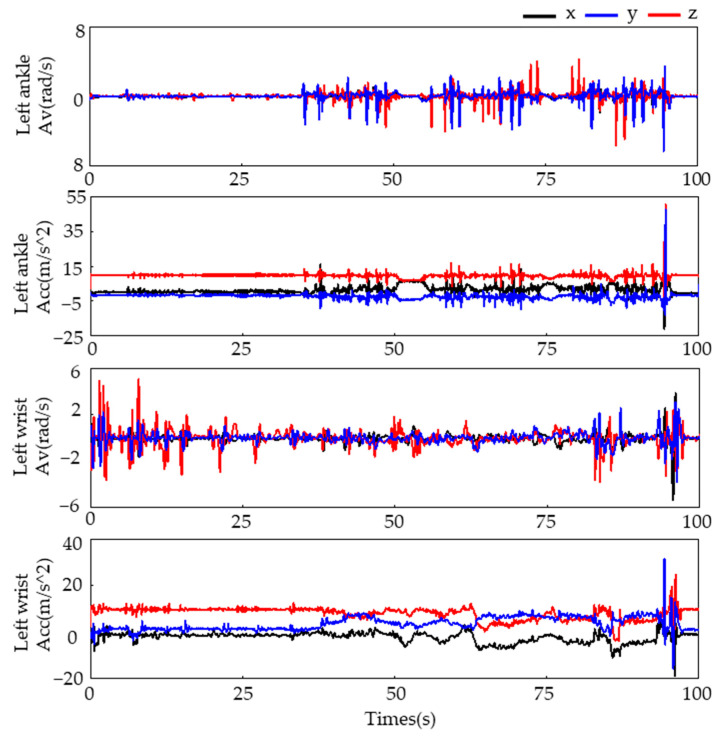
The collected motion data of the left wrist and left lower tibia.

To collect motion data, we attached four WMINS sensors to specific joints on the human body, as illustrated in Figure 1b. The motion data were collected at a sampling frequency of 30 Hz for a duration of 100 s. The three-axis acceleration and three-axis angular velocity data collected from the left wrist and left lower tibia are displayed in Figure 2.

### 2.2. VR Scene and Task Introduction

We designed a set of virtual scenarios to induce controllable states of acrophobia and explore two different movement states, acrophobia and non-acrophobia, in response to acrophobia. The scenarios were created using VR scenes and WMINS, and were centered around the psychological stress response of individuals to acrophobia environments or events in the real world. Our goal was to create a repeatable and easily operated stress induction paradigm in the laboratory environment, which would elicit subjects’ stress response under natural conditions while recording their movement state. The virtual scene mode was comprised of three main parts: the VR scenario, physical tactile stimulation, and data acquisition. The VR scenario consisted of a 60-story high-rise building designed in VR. Subjects were transported to the high-altitude scenario via an elevator and encountered a virtual wooden board extending outward from the high-altitude elevator. At the end of the wooden board, a high-altitude diving platform was designed, with four basketball hoops placed in front, back, left, and right of the wooden board, respectively. Additionally, two virtual tennis balls were designed in the rectangular basket at the end of the wooden board.

**Figure 3:**
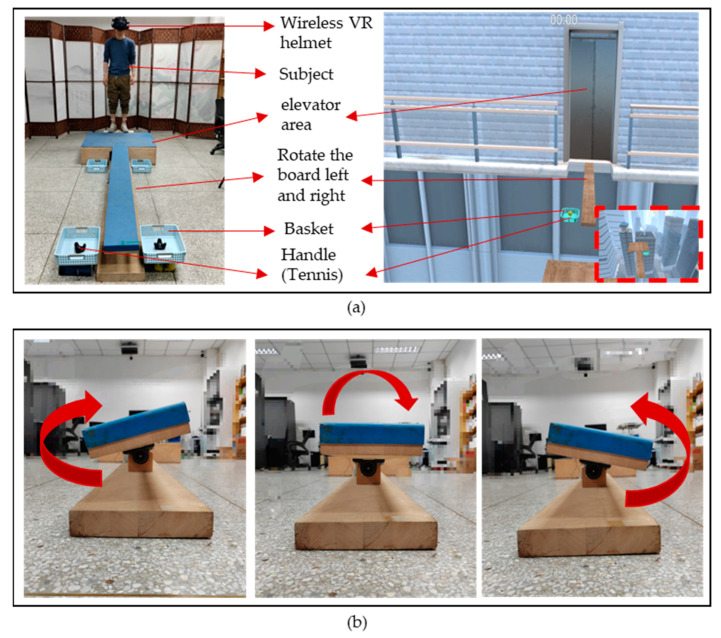
Introduction to the VR experiment scene. (**a**) The connection between the VR environment and the actual scene construction. (**b**) The plank turns left and right.

In addition to building a high-altitude scene in the virtual environment, we also constructed a real scene that was 25 cm above the ground, based on the virtual coordinates. The size of the plank and basketball hoops in the real scene were designed to be in a 1:1 ratio with the virtual scene, ensuring that the subjects could fully experience the VR scene in the helmet while maintaining safety during the experiment. To increase the realism of the VR scene, we incorporated a vibration module in the elevator area in the physical touch stimulation part. When the subject stood in the elevator area, the vibration module was activated when the elevator in the VR scene ascended. This allowed the subject to feel the vibration of the elevator ascending from the soles of their feet, simulating the ascent and descent of the high-altitude elevator in the virtual environment. Figure 3b shows the left and right swaying of the plank in the experimental scene. In the VR scene, the plank swayed left and right, so we designed a plank rotation mode with the same amplitude and a rotation frequency of 0.5 Hz. The maximum inclination angle of the plank was 16 degrees. When subjects walked on the plank, they could feel the realism of the VR scene. Two handles were placed in the end basket of the plank to simulate the tennis ball models in the VR scene. Throughout the experiment, the subject’s motion data were collected in real-time by the WMINS sensors and obtained and saved in real-time by connecting to the VR scene. Overall, our experimental design allowed us to create highly realistic and immersive experience for the subjects while collecting valuable data on their psychological and physiological responses in high-altitude environments and related anxiety disorders.

### 2.3. Acrophobia Scale

We utilized two assessment scales, the virtual reality scene quality assessment (VRSQ) [26] and the height interpretation questionnaire (HIQ) [27], to evaluate the subjects’ response to specific VR environments and their level of acrophobia, respectively. The VRSQ is a questionnaire that evaluates the level of dizziness and discomfort experienced by users in VR environments. It was developed in 2002 by Kenneth J. Miller et al. We also comprised 16 questions that describe the onset time, severity, and various possible symptoms of dizziness and discomfort. These questions aid evaluators in determining the degree of discomfort experienced by users in VR environments. The HIQ, developed jointly by American psychologists Gary R. Parker and Robert G. Stumpf, is a questionnaire consisting of 32 questions designed to assess acrophobia. The questionnaire is primarily divided into three parts: high-altitude experiences, high-altitude imagination, and behavioral avoidance. Subjects answer these questions based on their actual experiences, and the answer is selected on a five-level scale. By statistically analyzing the responses of subjects, an overall HIQ score can be obtained, with a higher score indicating a more severe level of acrophobia. The full HIQ scale has a Cronbach alpha of 0.87, meaning that the HIQ is a reliable instrument for measuring height interpretation. In summary, the HIQ is a valid psychometric tool with high reliability and validity that can help clinicians and psychologists understand the level of individual acrophobia and provide a valuable reference for the treatment and prevention of acrophobia.

### 2.4. Experimental Design

The procedure was as follows: prior to the test, the subjects complete the VRSQ and HIQ scale to assess their acceptance of the VR environment and to make a preliminary assessment of their acrophobia state. The subjects then enter the three-dimensional virtual scene using a wireless VR helmet and WMINS. They stand in the elevator area, where the vibration module is automatically activated to simulate vibration as the elevator rises and falls. When the virtual elevator reaches the 60th floor, the elevator door opens, and subjects exit the elevator area, as detected by the wireless VR helmet’s movement tracking. The movement of leaving the lift triggers a rotational movement of the board from side to side, while the participant has to balance on the rotating board at all times and walk to the end of the board, pick up the tennis ball in the rectangular basket at the end of the board, turn around, and walk back to the starting position of the board and place the tennis ball into the rectangular basket at the starting position. After placing the tennis ball in the rectangular basket at the start, they then continue to the end of the board and pick up an-other tennis ball and walk back to the start of the board and place the tennis ball in it. When the last tennis ball has been placed in the rectangular basket at the start position, the left/right switch will automatically turn off and the board will automatically re-turn to a horizontal position. At this point, the participant walks to the end of the board and jumps 40 cm onto a circular diving platform in front of them.

**Figure 4:**
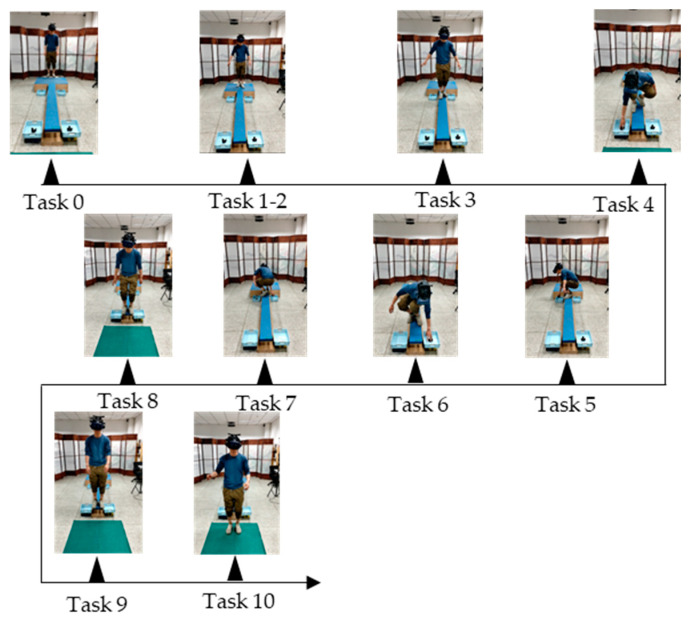
Eleven kinds of task action display under the actual VR acrophobia experiment.

For the purpose of data analysis, we divided the entire VR acrophobia experiment into three main stages: the rest adaptation stage, the board walking stage, and the jumping stage. These three stages were further divided into 11 tasks, as shown in Table 1. In the rest adaptation stage, subjects were divided into four task stages in the VR elevator environment, including resting and waiting in the elevator interior (task0), elevator ascent (task1), elevator door opening (task2), and elevator closing after subjects walked out of the elevator (task3). In the board walking stage, subjects were required to walk back and forth twice on a fixed frequency shaking board, pick up the tennis balls located at the end of the board and place them in the net located in the same direction at the beginning of the board. This stage included tasks 4 to 8. The jumping stage included the jump preparation stage (task9) and the final jump stage (task10). In each stage of the experiment, the movement status of the left and right wrist and the left and right lower tibia of the subjects were recorded and transmitted to the computer receiver in real time by WMINS. Figure 4 details the specific movements of subjects in the 11 tasks.

### 2.5. Acquisition of Experimental Data

A total of 31 subjects were recruited for the present study, and each participant completed 2 experiments. All subjects had no history of neurological disorders or photosensitive epilepsy, and furthermore, were in a normal physical state with basic limb movement abilities during the experiment. Prior to the experiment, subjects were correctly informed of the specific details of the experimental procedure, including the purpose, process, etc., and signed an informed consent form. By triggering each task end node in order among the 11 tasks, a complete VR acrophobia experiment was performed. Finally, 1 experiment can obtain 11 segments of movement data and generate movement data files for each participant for each experiment at key points of the limbs. Due to unexpected situations such as task failure or task timeout during the experiment, 43 usable sample data were obtained after data cleaning, including 24 male subjects and 19 female subjects, with all subjects aged between 23 and 30 years old. According to the HIQ questionnaire administered before and after the experiment to label the subjects, those with HIQ scores greater than 29 were classified as the acrophobia group and those with scores less than 29 were classified as the non-acrophobia group [28]. Finally, there were 16 HIQ scores greater than 29 and 27 scores less than or equal to 29 in the 43-point sample, which included 1 sample with a score of exactly 29.

**Figure 5:**
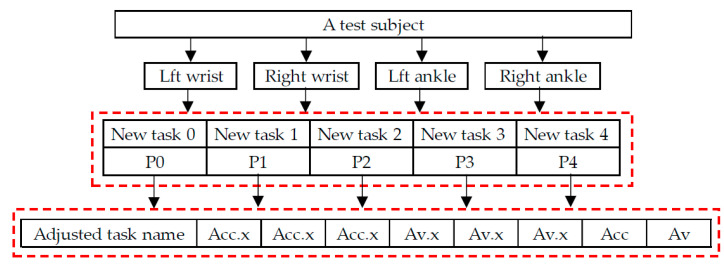
The adjusted experimental data composition display.

Considering that the speed at which the subjects perform the tasks in the experiment may vary greatly, hesitant behavior may occur in some tasks for subjects with acrophobia, while non-acrophobia subjects may quickly pass through the experiment, resulting in insufficient movement data for some tasks, making it difficult to obtain accurate quantitative features. Therefore, the 11 tasks were adjusted based on their nature and divided into 5 major tasks, as shown in Table 1. The adjusted Task 3 includes the entire process of picking up and putting down the ball, while Task 4 includes the entire motion data during the jumping period. As shown in Figure 5, the sample data for each subject contains movement data of four joints (right and left wrist and the left and right lower tibia) in the human body, with each joint movement data including five task types, with each data point in each task type including the adjusted task name, acceleration and angular velocity along three axes, and calculated three-axis combined acceleration and angular velocity.

## 3. Results

**Figure 6:**
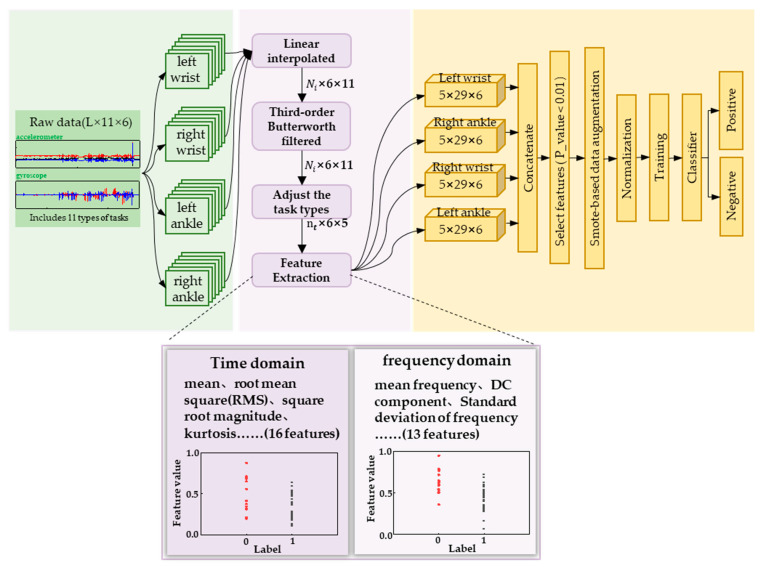
The general diagram of the motion data processing model system for scenes of acrophobia. $N_{i}$ indicates the data length of each of the 11 tasks, $n_{t}$ indicates the data length of each task after task adjustment, where i and t both indicate the number of tasks.

We divided the labeled motion data of the subjects into four types of joint data based on the attachment position of the sensor, namely left wrist data, right wrist data, left lower tibia data, and right lower tibia data. As illustrated in Figure 6, each joint data type corresponds to a 6-dimensional matrix under 11 task types. Since the motion under the 11 task types is continuous, we preprocessed the individual joint data by interpolating and low-pass filtering according to the data dimension. Then, we adjusted the 11 task types into 5 major task types according to Table 1, and extracted the features from the data to obtain a task-based feature matrix. Finally, we transformed the data to obtain four joint feature matrices. Based on this, sample data were transformed from raw motion data to four feature matrices, and the three-dimensional feature matrices were transposed and concatenated to obtain an overall feature matrix. The feature matrix was subjected to feature selection and over-sampling, and the processed data were fed into machine learning model for training and classification, ultimately achieving a stable and highly accurate acrophobia classification model.

### 3.1. Feature Analysis

We conducted a detailed feature analysis of the preprocessed data. During the experiment, subjects with acrophobia label generally took longer to perform the tasks compared to non-fearful subjects, particularly in T4, where fearful subjects had more hesitation time during the final jumping moments, and the jumping movements appeared discontinuous. In T0, the subjects were in a closed space elevator where the floor number increased to represent the elevator going up. For fearful subjects, some of them may have had fewer limb movements due to their inner fear. In T2, when the elevator door opened, the sudden appearance of a high-altitude environment could give subjects a visual shock, increasing the sense of reality and stimulating the subjects’ belief in the high-altitude environment. Fearful subjects would spend more time building their confidence after the elevator door opened, and most of them would be in a state of external calmness, rather than a natural movement state as observed in non-fearful subjects who could adapt quickly to the environment. In T3, fearful subjects walked more carefully on the wooden board and their walking steps became smaller and slower, resulting in poorer continuity of their walking movements. In addition, their balance will become worse due to the movement of the plank. During the actual experiment, they keep the center of mass and the plank on one side at all times by a slight outward extension of the arms and an up-and-down movement, while the subject will bend slightly to lower the center of mass and increase stability as they move. In contrast, non-fearful subjects had better balance and control over their body movements.

Based on the observed behavioral characteristics, we conducted feature analysis on preprocessed data in terms of three-axis acceleration and three-axis angular velocity. Feature extraction was performed in both time and frequency domains to obtain a comprehensive set of features. In the time domain [29], we extracted typical features such as mean, variance, maximum, and minimum values for each data dimension under different task types. In addition, we also obtained specific features such as impulse factor, skewness factor, and peak-to-peak. In total, we extracted 16 features in the time domain. In the frequency domain [30], we extracted 13 different types of features, including frequency center, RMS frequency, and DC component. Ultimately, each data dimension for different task types had a total of 29 features in both time and frequency domains. Table 2 provides detailed information on these 29 features. Finally, we split and combined the feature data according to the joint positions specified by WMINS, resulting in 4 joint positions with 5 × 29 × 6 feature matrices.

After feature data segmentation and solving, we obtained 3480 different feature types. However, the number of samples was only 43, much less than the number of feature types. To prevent overfitting in the subsequent classification model, we performed feature selection using *p*-values, a statistical method [31]. *p*-value is a probability value obtained from statistical analysis, which represents the probability of observing the results or more extreme results under the null hypothesis. When the *p*-value is less than the set significance level (e.g., 0.05), the null hypothesis is rejected, and the observed results are considered significant, indicating that the differences observed in the sample may not be due to chance. When the *p*-value is greater than the set significance level, the null hypothesis cannot be rejected. Here, we have labeled the acrophobia group “0” and the non-acrophobia group “1”. Similarly, the features solved for the acrophobia group will be labelled “0” and the features solved for the non-acrophobia group will be labelled “1”, and finally, the *p*-values for each class of features will be solved directly for that class of features based on these feature values and the corresponding labels. We sorted the WMINS under each joint in ascending order according to their *p*-values and selected the features whose *p*-values were less than 0.02. We finally obtained 88 data features and replaced the original motion data with these features to represent the motion state of each subject. These 88 features were used as the original feature data set for building the classification model.

### 3.2. Sample Balancing

As there was an imbalance in the sample data between non-acrophobia and acrophobia samples, which could lead to a decrease in model generalization and prediction accuracy, K-means smote was used to oversample the sample data [32]. K-means smote is an improved oversampling method used to address the issue of class imbalance in classification problems. K-means smote uses a weight scheme based on the K-nearest neighbor algorithm to control the weights between different samples to avoid oversampling between different clusters. The 88 features were oversampled using K-means smote for each feature dimension, resulting in an increase in the data weight of each feature type from 43 to 56 samples. With 88 feature types representing 1 sample, a total of 56 samples were obtained, with 13 additional acrophobia feature samples.

### 3.3. Acrophobia Classification Model

We designed a voting classification algorithm specifically for the acrophobia and non-acrophobia movement data. Voting is an ensemble learning-based classification algorithm [33] that uses a majority vote to determine the final classification. We used KNN [34], extra trees, random forest [35], SVM [36], and logistic regression [37] as the five classifiers for model ensemble. First, we divided the dataset into a training set and a test set. During the training phase, we used the training set data to train the five classifiers and obtained five well-trained models. Finally, we input the test set data into each well-trained model for classification, obtaining the classification results of each model. Then, we aggregated the classification results of each model and used hard voting to determine the final classification result. Specifically, for each test sample, we counted the classification results of the five classifiers, and selected the class with the most votes as the final classification result. The voting classification algorithm we designed combines the advantages of five machine learning models and has better classification performance for acrophobia and non-acrophobia movement data.

### 3.4. Evaluation Metrics

In order to comprehensively evaluate the classification performance of our algorithm models, we used four quantitative evaluation parameters: accuracy, recall, precision, and F1-score [38]. Accuracy measures the proportion of correctly predicted samples to the total number of samples, and is a good indicator of model performance when the proportion of positive and negative samples is relatively balanced. Recall measures the proportion of correctly predicted positive samples to the actual number of positive samples, reflecting the model’s ability to detect positive samples. Precision measures the proportion of correctly predicted positive samples to the total number of predicted positive samples, indicating the proportion of true positives in the predicted positive samples. F1-score is the harmonic mean of precision and recall, providing a comprehensive measure of the model’s performance. The higher the F1-score, the better the performance of the classification model.

(1) $$\mathrm{precision}=\frac{\mathrm{TP}}{\mathrm{TP}+\mathrm{FP}}$$

(2) $$\mathrm{recall}=\frac{\mathrm{TP}}{\mathrm{TP}+\mathrm{FN}}$$

(3) $$\mathrm{accuracy}=\frac{\mathrm{TP}+\mathrm{TN}}{\mathrm{TP}+\mathrm{FP}+\mathrm{TN}+\mathrm{FN}}$$

(4) $$F1=\frac{2\times \mathrm{Precision}\times \mathrm{Recall}}{\mathrm{Precision}+\mathrm{Recall}}$$

These evaluation metrics can be defined by Equation (1) to Equation (4). Here, TP represents the number of positive samples that the model predicted as positive, FP represents the number of negative samples that the model predicted as positive, FN represents the number of positive samples that the classification model predicted as negative, and TN represents the number of negative samples that the classification model predicted as negative.

### 3.5. Experimental Platform and Model Setting

We employed a computer equipped with an Intel Core i5-11400 processor, 16 GB of RAM, and an NVIDIA GeForce RTX 3060 GPU for machine learning training and testing. The computer runs on the Windows 10 operating system and utilizes the Python programming language for algorithm implementation.

We utilized leave-one-out cross validation (LOOCV) to evaluate the classification performance of our model, a commonly used cross-validation method in machine learning. LOOCV trains and evaluates the model on each sample as the validation set, ultimately averaging the evaluation results for each sample, providing an objective evaluation of model performance. After dividing the dataset into training and test sets using the leave-one-out method, we standardized the training set by normalizing its feature data, ensuring that the distribution of feature data had similar scales and ranges to improve the model’s accuracy. The standardization formula (Equation (5)) was applied to the training set and then to the test set to normalize the test set in the same manner.

(5) $$x_{n}=\frac{x_{n}-\mathrm{mean}\left(x\right)}{\mathrm{std}\left(x\right)}$$

To verify the effectiveness of our designed voting method, we constructed 10 typical machine learning models, including k-nearest neighbor, decision tree, random forest, naive Bayes classifier, support vector machine, ensemble learning, logistic regression, multi-layer perceptron, Gaussian naive Bayes, and extremely randomized trees. We used the leave-one-out cross-validation method to evaluate each model, ensuring the most objective evaluation of model performance.

### 3.6. Results and Analysis

**Figure 7:**
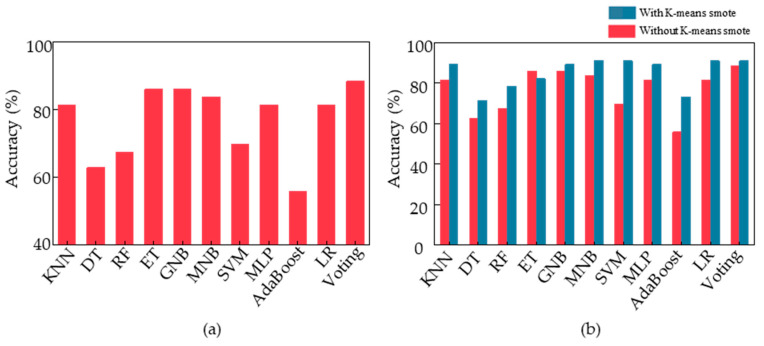
Classification accuracy of each model. (**a**) Classification accuracy of each model obtained by training the sample data without K-means smote. (**b**) Comparison of classification accuracy obtained by training the sample data with and without K-means smote.

We evaluated our designed voting algorithm using feature sample data that had not undergone K-means smote through 43 cycles of leave-one-out cross validation, resulting in a final classification accuracy of 88.37%. Additionally, we fed the same data processed through the same procedure into the other 10 machine learning models we built [39]. Figure 7a displays the final classification accuracies of these 11 models, with our voting ensemble model achieving the highest accuracy of 88.37%, followed by ET and GNB at around 86%. Conversely, DT, RF, and AdaBoost had poor classification performance, with an accuracy of only approximately 60%. Among them, AdaBoost was the worst-performing classification model with an accuracy of only 55.8%.

To improve the performance of the classification model for acrophobia based on motion data, we applied K-means smote to the feature samples, oversampling the data and feeding it into the voting ensemble model we designed. This resulted in a classification accuracy of 91%. To verify the effectiveness of K-means smote, we fed the new feature samples into 10 other models and compared the accuracy with and without oversampling. Figure 7b illustrates the changes, showing that the K-means smote oversampled data are of higher quality and lead to significant improvements in classification accuracy across various models, including LR, MNB, SVM, and voting, with all achieving a classification accuracy of 91%. The most significant improvement was observed in the AdaBoost model, which achieved a final classification accuracy of 73.2%, an increase of 17.4% from the initial accuracy. Therefore, K-means smote can optimize motion data for acrophobia classification and improve the overall performance of the model at the data level.

**Figure 8:**
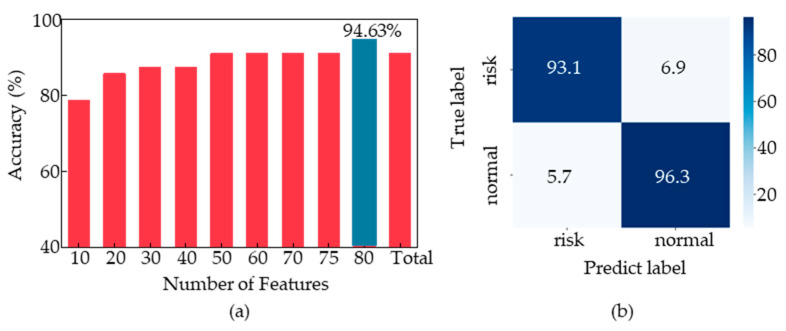
(**a**) Classification effect of voting model with different combinations of feature data. (**b**) Confusion matrix with the optimal combination of feature data.

Building upon this, we explored the impact of different feature combinations on the performance of the model among 88 features. We reordered the 88 features based on their *p*-values in ascending order and selected the top 10, 20, and so on, at intervals of 10. We thus obtained eight new sets of feature combinations and fed them into the voting ensemble model we designed. We obtained the optimal feature set for each feature data combination. As shown in Figure 8a, we compared the classification accuracy of the voting ensemble model under the 88 features with the other 8 data combinations and found that the classification accuracy reached 94.63% for the top 80 feature combination, which was the highest among all combinations. To further validate the results, we selected the top 75 features with the smallest *p*-values for voting ensemble model training and achieved an accuracy of 91.7%. Figure 8b shows the confusion matrix of the voting ensemble model, with a classification accuracy of 96.3% for non-fear-of-heights samples and 93.1% for fear-of-heights samples.

**Figure 9:**
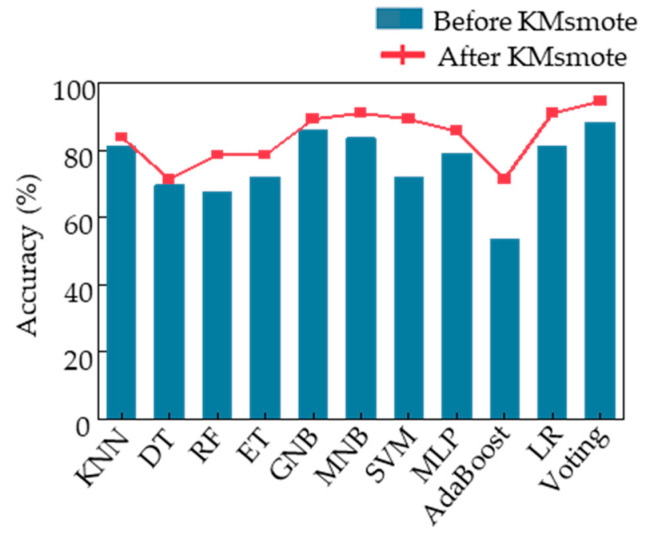
Classification accuracy of each model under Top 80 feature data samples.

The classification accuracies of the top 80 samples were fed into 10 different machine learning models, and the results are shown in Figure 9. The blue bars represent the classification accuracies of the models using the original top 80 samples without oversampling, while the red line represents the classification accuracies of the models using the top 80 samples after K-means smote oversampling. It can be observed that the Voting algorithm model designed in this study achieved the highest classification accuracy for both the original and oversampled feature samples.

Table 3 presents the specific performance of different algorithm models, and the optimal voting ensemble model achieved a precision of 96.2%, a recall of 92.8%, and an F-score of 94.5%. The precision and F-score were the highest among all models, indicating that the voting ensemble model had the best comprehensive ability to predict non-fearful and fearful samples. In terms of classification accuracy, both the LR model and MNB model had the second-best classification ability, with the same accuracy of 91.07%. However, the precision of the MNB model was 92.59%, while that of the LR model was 88.88%, indicating that the MNB model had a better classification effect on non-fearful samples. In terms of recall, the scores of the 2 models were exactly opposite, with the LR model achieving a score of 92.3% and the MNB model only achieving 89.2%. This indicates that the LR model has a stronger ability to identify non-fearful samples compared to the MNB model.

**Figure 10:**
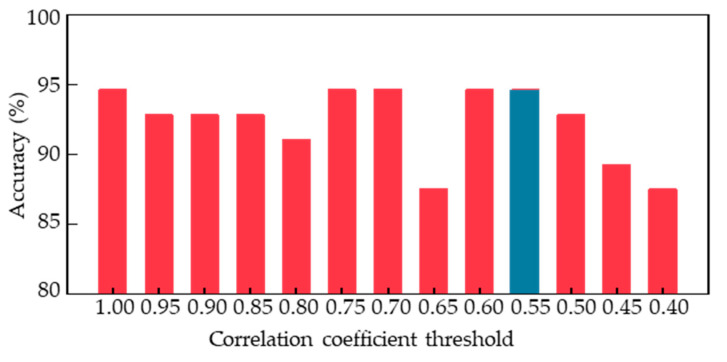
Classification accuracy of feature combinations in voting ensemble models with different correlation coefficient thresholds.

In order to explore the optimal combination of human body limb motion features for acrophobia classification and obtain a superior classification model, further analysis was conducted on the top 80 features, as the number of features was too high compared to the final 56 samples. Specifically, we computed the cross-correlation coefficients [40] between different features and determined a series of threshold values to remove redundant features. By setting the correlation coefficient threshold to 0.9, we removed one of the features with a correlation coefficient greater than 0.9, resulting in 48 feature combinations. Figure 10 shows the classification performance of the voting ensemble model trained on these new feature combinations with different correlation coefficient thresholds ranging from 0.4 to 1. We found that the model achieved the best classification accuracy when the correlation coefficient threshold was set to 1, 0.75, 0.7, 0.6, or 0.55, with all accuracies reaching 94.6% through the leave-one-out cross validation. When the threshold was set lower than 0.55, the model’s performance began to decline. Therefore, 0.55 was determined as the minimum optimal threshold, resulting in a reduced number of feature types to only 21.

## 4. Discussion

**Figure 11:**
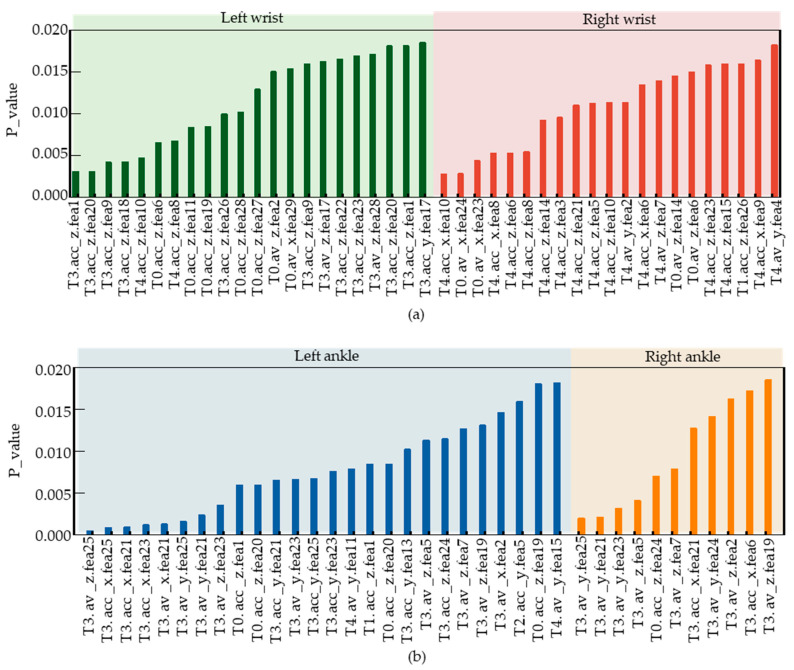
Feature types and corresponding *p* values after feature selection. (**a**) Type of feature selected at the wrist joint and the corresponding *p*-value. (**b**) Type of feature selected at the lower tibiae and the corresponding *p*-value.

In this study, a high classification accuracy model of acrophobia and non-acrophobia was developed; on the basis of that, it was revealed that acrophobic and non-acrophobic populations possess different motor patterns under high-altitude conditions. Figure 11 illustrates the selected feature series after feature selection. T3.av_z.fea25 is the feature with the smallest *p* value, representing feature 25 of the Z-axis angular velocity in the left lower tibia movement data during the T3 task phase. The meanings of the other feature symbols on the horizontal axis are the same as described above. Figure 11a shows the selected left and right wrist features after screening, with a total of 22 features for the left wrist and 21 features for the right wrist. Since the number of features in the left and right wrists is almost equal, the contribution weight of the left and right wrists is equal in the classification of the acrophobia and non-acrophobia labels. Figure 11b shows the selected features in the left and right lower tibia movements, with 26 features for the left lower tibia and 11 features for the right lower tibia, indicating that the left lower tibia contributes more weight to the classification of acrophobia and non-acrophobia due to the asymmetry of lower limb movements in the VR acrophobia experiment. This may be due to the limited walking space on the wooden plank and the subjects’ high-altitude state, which causes the lower limbs to move continuously in a single direction, leading to differences in lower tibiae movements. Figure 11b reveals that the left leg is the main force-bearing part, and the right leg is the driven part during walking for both acrophobia and non-acrophobia subjects. Moreover, the T3 task phase is the main walking stage throughout the experiment, which explains why the T3 task phase features account for over 75% of the lower tibiae features, while only about 30% of the wrist features. In conclusion, there are 43 selected features in the wrist and 37 in the lower tibiae, indicating that the upper and lower limbs have a similar weight in acrophobia and non-acrophobia labels.

In addition, we compared our results with those of similar related studies. Hu et al. collected EEG data from subjects in a virtual environment and used deep learning networks to build a 4-classification model, achieving an accuracy of 88.77% [41]. R. Zheng et al. analyzed fear by acquiring EMG, Pupil, and ECG signals from subjects, and built a classification model combining deep learning and machine learning, achieving a binary classification accuracy of 93.93%. Other researchers have either used single or multi-modal physiological signals for subject-related analysis in virtual environments and classified them using ML and DL. These detailed results are shown in Table 4. Among these classifications, our system model achieved the highest classification accuracy of 94.6% using sparse joint data. The 21 selected features significantly reduced the training data required while improving the model’s generalizability. This will greatly enhance training efficiency when analyzing large datasets in the future.

## 5. Conclusions

By setting up a series of movement tasks in a virtual reality high-altitude scenario, this study investigated human motion characteristics, and based on these motion characteristics, an in-depth study was conducted to establish a classification model with high accuracy, confirming that there are differences in the movement patterns of people with and without acrophobia in high-altitude environments. This research will enable rapid screening of both acrophobic and non-acrophobic people, and additionally, will provide quantitative profiling that can be used in the future to provide more targeted training guidance for aviation personnel, as well as providing rapid training results.

In the future, we will continue to optimize WMINS to obtain more data on key human joint movements to reconstruct human posture, improve the VR high-altitude scenario while ensuring safety, and explore the contribution of limb movements in different parts of the human body to the correct classification of acrophobia. We will achieve the fine-grained classification of acrophobia and non-acrophobia under different weights, thereby exploring the correlations between limb movement characteristics and human fears.

## Figures and Tables

**Table 1:** Specific task division of VR acrophobia experiment.

Task Name	Task Content	Adjusted Task Name
Task 0	Rest and wait stage	New task 0 (T0)
Task 1	Elevator ascending stage	New task 1 (T1)
Task 2	Elevator opening stage	
Task 3	Elevator closing stage	New task 2 (T2)
Task 4	Pick up the ball Ⅰ	New task 3 (T3)
Task 5	Release the ball Ⅰ	
Task 6	Pick up the ball Ⅱ	
Task 7	Release the ball Ⅱ	
Task 8	Go to the end of the single wooden bridge	
Task 9	Pre-jump preparation stage	New task 4 (T4)
Task 10	Jump to the specified position stage	

**Table 2:** Selected time-domain and frequency-domain feature names and corresponding notations.

Symbol	Feature
fea1	mean
fea2	root mean square (RMS)
fea3	square root magnitude
fea4	absolute average
fea5	skewness
fea6	Kurtosis
fea7	variance
fea8	max
fea9	min
fea10	peak-to-peak
fea11	form factor
fea12	crest factor
fea13	impulse factor
fea14	margin factor
fea15	skewness factor
fea16	kurtosis factor
fea17	Mean frequency
fea18	Standard deviation of frequency
fea19	the degree of dispersion or concentration of the spectrum
fea20	DC component
fea21	frequency center
fea22	the degree of dispersion or concentration of the spectrum
fea23	RMS frequency
fea24	Indicates a change in the position of the main frequency band
fea25	Indicates a change in the position of the main frequency band
fea26	the degree of dispersion or concentration of the spectrum I
fea27	the degree of dispersion or concentration of the spectrum II
fea28	the degree of dispersion or concentration of the spectrum III
fea29	the degree of dispersion or concentration of the spectrum IV

**Table 3:** Classification results of different algorithmic models in top 80 feature sample data.

Models	Accuracy	Precision	Recall	F-Score
KNN	0.83929	0.85185	0.82143	0.83636
DT	0.71429	0.62963	0.73913	0.68000
RF	0.78571	0.74074	0.80000	0.76923
ET	0.78571	0.77778	0.77778	0.77778
GNB	0.89286	0.88889	0.88889	0.88889
MNB	0.91071	0.92593	0.89286	0.90909
SVM	0.89286	0.92593	0.86207	0.89286
MLP	0.85714	0.85185	0.85185	0.85185
AdaBoost	0.71429	0.62963	0.73913	0.68000
LR	0.91071	0.88889	0.92308	0.90566
VOTE	0.94643	0.96296	0.92857	0.94546

**Table 4:** Comparison with other stress scenario classification models.

Study	Signals	Method	Class	Accuracy
Hu et al. [41]	EEG	DL	4	88.77%
Bălan et al. [39]	EEG, HR, GSR	ML, DL	2-\4-choice scale	89.50%\42.50%
Salkevicius et al. [42]	GSR, BVP, skin temperature	SVM	4	86.30%
Zhang et al. [43]	GSR	BP	2	86.70%
R.Zheng et al. [44]	EMO, Pupil, ECG	ML, DL	2	93.93%
Our method	body motion data	EL	2	94.60%

## Data Availability

The data are not publicly available due to the relevant project regulations.
